# Assessment and Monitoring of the Wound Micro-Environment in Chronic Wounds Using Standardized Wound Swabbing for Individualized Diagnostics and Targeted Interventions

**DOI:** 10.3390/biomedicines12102187

**Published:** 2024-09-26

**Authors:** Julian-Dario Rembe, Waseem Garabet, Jan-Wilm Lackmann, Sadaf Alizadehrahrouei, Matthias Augustin, Joachim Dissemond, Wiebke Ibing, Karl Köhrer, Klaus Pfeffer, Anna Rommerskirchen, Sebastian Alexander Scharf, Tobias Wienemann, Thorsten Wachtmeister, Hubert Schelzig, Ewa Klara Stuermer

**Affiliations:** 1 Department for Vascular and Endovascular Surgery, University Hospital Duesseldorf (UKD), Heinrich Heine University Duesseldorf, 40225 Duesseldorf, Germany; waseem.garabet@med.uni-duesseldorf.de (W.G.); sadaf.alizadehrahrouei@med.uni-duesseldorf.de (S.A.); wiebke.ibing@med.uni-duesseldorf.de (W.I.); hubert.schelzig@med.uni-duesseldorf.de (H.S.); 2 Cologne Excellence Cluster for Cellular Stress Responses in Aging-Associated Diseases (CECAD), University of Cologne, 50923 Cologne, Germany; jan-wilm.lackmann@uni-koeln.de; 3 Institute for Health Services Research in Dermatology and Nursing Professions (IVDP), University Medical Center Hamburg-Eppendorf (UKE), 20251 Hamburg, Germany; m.augustin@uke.de; 4 Department of Dermatology, Venereology and Allergology, Essen University Hospital, 45147 Essen, Germany; joachim.dissemond@uk-essen.de; 5 Biological and Medical Research Center, Genomics and Transcriptomics Laboratory (GTL), Heinrich Heine University Duesseldorf, 40225 Duesseldorf, Germany; koehrer@uni-duesseldorf.de (K.K.); thwac001@hhu.de (T.W.); 6 Institute for Medical Microbiology and Hospital Hygiene, Heinrich Heine University Duesseldorf, 40225 Duesseldorf, Germany; klaus.pfeffer@hhu.de (K.P.); anna.rommerskirchen@med.uni-duesseldorf.de (A.R.); sebastianalexander.scharf@med.uni-duesseldorf.de (S.A.S.); tobias.wienemann@med.uni-duesseldorf.de (T.W.); 7 Clinic and Polyclinic for Vascular Medicine, University Heart and Vascular Center, University Medical Center Hamburg-Eppendorf (UKE), 20251 Hamburg, Germany; e.stuermer@uke.de

**Keywords:** chronic wound, personalized medicine, wound swabbing, wound micro-environment, Wound-OMICS, diagnostics, proteomics, metagenomics, sRNA-seq, whole genome sequencing

## Abstract

**Background/Objectives:** Patient-specific diagnostic and therapeutic approaches are important in the care of people with chronic wounds. The heterogeneity of underlying disease profiles and the diversity of the wound micro-environment make generalized approaches difficult. While high-throughput molecular diagnostic methods are increasingly widespread and available, the analysis of objective biomolecular disease patterns has not found its way into everyday wound management. The aim of this study is to evaluate the use of wound swab samples for the analysis of biomarkers and disease patterns in people with chronic wounds. **Methods:** A sample cohort from the multicenter “Wound-BIOME” project was analyzed. The project aims to comprehensively investigate the local micro-environment of chronic wounds of various entities, healing tendencies and regeneration stages at the biomolecular level. A sample collection and handling protocol suitable for everyday use was tested and evaluated regarding feasibility for multiplex immunoassay, proteomics, small RNA sequencing (miRNA) and metagenome analyses (microbiomics). **Results:** It could be shown that standard wound swabs are well-suited for the analysis of the complex wound micro-environment using various high-throughput methods. Despite the sample heterogeneity, the quality was adequate to analyze biomolecular patterns. **Conclusions:** Initial analyses of protein signatures, microbial wound communities and miRNA patterns show promising results for future individualized diagnostics and targeted interventions.

## 1. Introduction

The diagnosis and management of people with chronic wounds remain a challenge in daily clinical practice [1]. In particular, the diversity of underlying pathologies and the variable metabolic responses of human tissues pose a challenge. Despite the complexity of evaluating the current state of a chronic wound as well as its therapeutic need, diagnostic methods and monitoring modalities of chronic wound management remain primarily clinical. Healthcare providers need to rely on experience and visual/tactile inspection, clinical evaluation and microbiological cultures, which may not provide comprehensive information on the wound micro-environment and healing progress. The decision-making process regarding local wound treatment is therefore subjective and prone to qualitative fluctuation based on individual experience and expertise. While individual expertise is undoubtedly relevant in managing a heterogenous problem such as chronic wounds, objective steering parameters to allocate necessary interventions and new treatment modalities to wound patients that objectively benefit from such are long called for to improve chronic wound management. However, the ongoing development and improved availability of molecular analysis and diagnostic methods are opening up new opportunities for the systematic characterization of wounds at the cellular and molecular levels. High-throughput methods such as various multiplex and OMICS approaches (e.g., proteomics, transcriptomics, microbiomics) are able to address the heterogeneity and complexity of the wound micro-environment [2,3,4,5].

In this context, the study of the biomolecular signature of chronic wounds is becoming increasingly important [6]. Acute and chronic wounds differ not only in their healing times but also in their molecular composition during the healing process. A number of mechanistic problems must be addressed, including prolonged inflammation [7], a destructive proteolytic environment [8], chronic infection with biofilm formation [9] and dysregulated cellular phenotype shifts [10]. Furthermore, these processes cannot be considered in isolation, as they are synergistically and antagonistically intertwined. The identification of specific biomarker patterns associated with the wound entity, healing trajectory or dysregulated processes could therefore constitute an important contribution to targeted and personalized wound care. However, this requires appropriate, readily available and easy-to-use access to testable material. Wound swabbing offers such an approach, as it is a minimally invasive, universally available and commonly used option to extract a diverse array of biomolecules from the wound micro-environment, including proteins, DNA, RNA, microorganisms and metabolites [11,12,13]. Wound swabs have already been extensively utilized in microbiological diagnostics, providing valuable diagnostic and daily therapeutic information [13].

In this study, we examine the potential for extending the use of wound swabs to other diagnostic areas. Our objective is to capture and analyze the biomolecular signatures and patterns of acute and chronic wounds. We present a highly suitable method for sample collection and processing suitable for everyday use and the results of initial feasibility analyses regarding material quantity, quality and basic downstream analyses. In this context, we highlight potential clinical applications of these diagnostic options, including prediction of wound healing progression, monitoring of treatment outcomes and potential development of new therapeutic approaches. This work is helping to deepen our understanding of the molecular mechanisms behind wound healing and, in the long term, could lead to improved and more targeted care for people with acute and chronic wounds.

## 2. Materials and Methods

### 2.1. Study Design and Recruitment

This study serves as the foundation for the overarching “Wound-BIOME” project and was primarily designed to assess the viability of a standardized, reliable sample acquisition and processing system for wound swabs for high-throughput biomolecular applications. The “Wound-BIOME” project (“Breakdown, Identification and Observation of the Micro-Environment in acute and chronic Wounds”) is a multicenter project with the objective of developing a more comprehensive understanding of the biomolecular signatures associated with the wound healing process. To achieve this objective, a range of modern diagnostic analysis methods are employed, including OMICS, next-generation sequencing (NGS), multiplex, hyperspectral imaging (HIS) and pH measurements. These are used to better characterize the complex environment of chronic, non-healing wounds, with the aim of providing insights for more objective, individual and targeted diagnostics and therapy.

**Figure 1:**
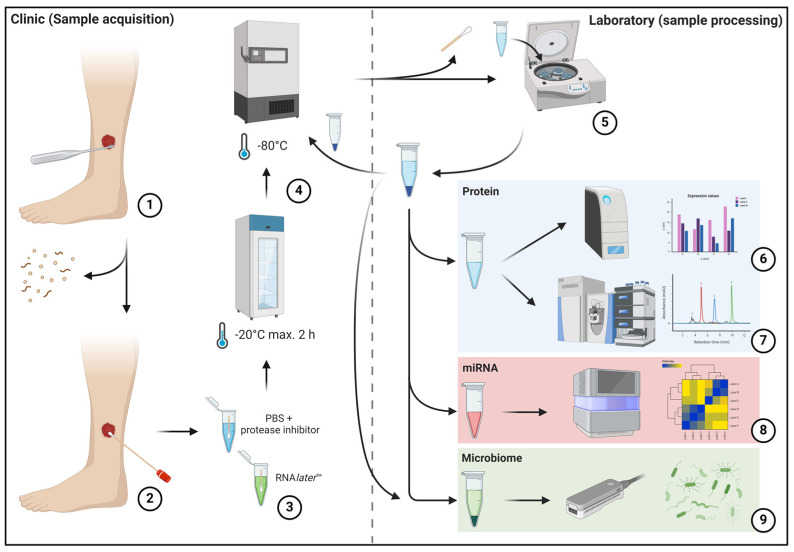
The left half of the image depicts the sample acquisition process in the clinical area, while the right half depicts sample preparation in the laboratory. The initial mechanical debridement of the wound bed is performed to remove excessive debris and dressing residues (1). This is followed by swabbing using FLOQSwab^®^ swabs (2) according to the “Essen Rotary” technique [14] and insertion into prepared transport media (3). Subsequently, the samples are frozen at a temperature of at least −20 °C within two hours and at a temperature of at least −80 °C within eight hours (4). This is followed by later sample processing, which involves removing the swab, pelleting cellular and debris components, and aliquoting the sample material for further analysis (5). The pelleted cell and debris fraction is frozen again at −80 °C for subsequent analyses. Subsequently, downstream analyses of the respective samples are conducted using multiplex immunoassay (6), mass spectrometry-based proteomics (7), small-RNA sequencing (8) and metagenome sequencing (9).

In this initial methodological feasibility study, protocols were developed and tested for the preparation of wounds, the collection of swabs and the further processing, storage and preparation of samples. The objective of establishing a standardized protocol is to minimize variance in sample collection and processing, thereby reducing the potential to introduce bias into analysis results due to technical handling. Concurrently, the sampling process must be straightforward and sufficiently robust to be utilized outside university medical centers, such as outpatient settings, under varying circumstances comparable to regular laboratory tests. Therefore, a simple, standardized sample collection process was devised that can be easily integrated into everyday clinical practice and can be carried out regardless of location (Figure 1).

The study design was observational, non-interventional and a multicenter cohort. The sample cohort represents a subgroup of the overall collective of the “Wound-BIOME” project, which aims to include samples from 142 patients with seven different entities. A multicenter design with multiple recruiters at different sites was deliberately employed from the outset in order to detect and adjust for differences in sample collection (despite the use of a uniform protocol) at an early stage. This approach ensures the collection of samples that are consistently stable. Due to the observational study design, the inclusion and exclusion criteria were formulated in a less restrictive manner to generate an uninfluenced, broad representation of the wound micro-environment (Table 1 shows the inclusion and exclusion criteria). All patients who were recruited were informed in detail in advance about the content and objectives of this study, including data protection aspects. Written informed consent was obtained from all participants prior to their involvement in this study.

Initial feasibility analyses were conducted on sample cohorts isolated from the total collective of patients recruited in the “Wound-BIOME” project. The objective was to evaluate sample preparation procedures, quality, quantity and suitability of the samples obtained through the workflow for biomolecular analysis methods. A total of at least 12 samples were obtained for each of the following analyses: immunoassay, proteomics and sRNA-seq, with six samples being collected for metagenomics. Furthermore, clinical and demographic data were collected in conjunction with the aforementioned analyses. This entailed the collection of additional data, including age, gender, wound size, wound entity and duration, among other variables. Furthermore, the wounds were evaluated by a healthcare professional at the time of sampling regarding their healing tendency, current healing phase, and clinical microbial load/infection. This was performed to enable subsequent analyses to be correlated with the clinical assessment. For this first exploratory purpose, variables such as healing status (healing vs. non-healing) were solely based on the clinical assessment of the primarily treating physician at the time of sample collection based on the current clinical trajectory. Table 2 provides an overview of the sample cohort analyzed.

Prior to the commencement of the study presented here, ethical approval was obtained for the overarching “Wound-BIOME” project, which includes the study in question. The principal ethics approval was granted by the Ethics Committee of Witten/Herdecke University (No. 11/2018). Subsequent study centers were also granted ethical approval by the relevant local ethics committees in Essen (No. 18-8432-BO), Hamburg (No. PV5883) and Duesseldorf (No. 2020-1012).

### 2.2. Sample Collection Process

#### 2.2.1. Swabs

Swabs were collected from people with acute and chronic wounds using FLOQSwabs^®^ (Copan Diagnostics Inc., Murrieta, CA, USA). The swabs comprise a solid, molded plastic applicator shaft and an outer nylon fiber structure sprayed onto the plastic shaft. These swabs are the most commonly used in microbiological diagnostics and have also been employed in earlier studies [12]. One advantage of these swabs over other types, such as cotton swabs, is that they prevent the sample from being firmly enclosed and have a strong capillary effect, which keeps the sample close to the surface and allows it to be easily released into suitable transport media. The swabs were individually wrapped and free of human DNA and RNA. The swabs were designed with a predetermined breaking point at 20 mm, allowing for the sample-wetted head to be easily broken off into a sample container with transport medium.

#### 2.2.2. Sample Container and Transport Medium

Standard Eppendorf tubes (1.5 mL; Eppendorf SE, Hamburg, Germany) were utilized as sample containers, which were pre-filled with 1 mL of an appropriate transport medium. For samples intended for the analysis of proteins, phosphate-buffered saline (PBS) with a universal protease/phosphatase inhibitor (PPC1010; Sigma-Aldrich, Merck KGaA, Darmstadt, Germany) was utilized as the transport medium. For analysis of RNA and DNA, samples were transported in RNAlater™ (Thermo Fisher Scientific Inc., Waltham, MA, USA) to stabilize and protect against degradation. The sample containers were prepared in advance and stored at 7 °C in a refrigerator until required.

#### 2.2.3. Sample Storage

Following the acquisition of the sample, the collected sample, including the swab tip, was frozen to a temperature of at least −18 °C to −20 °C within a maximum of two hours and to −80 °C within 24 h at the latest. This procedure was carried out irrespective of the intended downstream analysis (proteins, RNA, DNA). Samples were transported on ice or, in the case of longer distances, on dry ice from recruiting centers to the place of analysis in cooperating facilities in order to maintain the integrity of the cold chain. Special attention was paid to minimize the number of freeze–thaw cycles to reduce the risk of protein and nucleic acid (RNA/DNA) instability.

#### 2.2.4. Swabbing Technique and Collection Protocol

To minimize variability between recruiting centers and individuals when collecting the swab samples, a precise protocol for the sample collection procedure was established prior to this study. The swabs, sample containers, transport media and sample storage procedures were delineated in this protocol. Table 3 provides a brief overview of the sample collection procedure, including critical points.

The “Essen Rotary” [14] swabbing technique was selected, as it allows for the entire wound surface to be sampled, thus providing a comprehensive overall signature. Furthermore, this technique is widely used and well-known in German-speaking countries. The entire surface of the wound is swabbed in concentric circles, commencing from the exterior and progressing to the interior. The swab head is rotated repeatedly until the swab is visibly saturated. Following the removal of the sample, it was placed in the designated transport containers and, once appropriately labeled, frozen at −80 °C until further processing was required, dependent on the designated analyses.

### 2.3. Sample Preparation for Downstream Analysis

#### General Sample Processing

For subsequent processing of the wound swab samples, a single thawing/defrosting cycle was permitted, during which the swab tip remained within the sample and was subsequently removed, coarse cellular and detrital components were pelletized, and the samples were aliquoted into four aliquots of 210 µL each for planned downstream analyses.

Briefly, the samples were thawed slowly on ice (2–8 °C) over a period of two hours. Subsequently, the samples were vortexed with the remaining swab tip for three 5 s cycles to achieve the highest possible release of analytes from the swab into the transport medium (95–98% of bound analytes, according to the manufacturer). Subsequently, the swab tip was removed with sterile tweezers and rolled out once more over the interior of the sample vial. Subsequently, the swab tip was discarded, and the sample was vortexed once more for five seconds to achieve homogenization. Subsequently, two centrifugation steps were employed to remove cellular and microbial detritus, as the focus of the analysis was on the extracellular components and compartments. In the initial step, the samples were subjected to centrifugation at 10,000× *g* for 15 min at 4 °C (for RNA/DNA: 500× *g* for 15 min). Subsequently, the supernatant was transferred to a new sample tube without disturbing the pellet formed at the bottom, and centrifugation was performed again at 10,000× *g* for 15 min (for RNA/DNA: 2000× *g* for 10 min). Supernatant was divided into four distinct tubes, each of a different color to code for subsequent analyses, and the remaining cell and debris pellet was also frozen at a temperature of −80 °C for subsequent analysis. This process was performed identically for the protein and RNA/DNA-designated samples, thereby minimizing the number of freeze/thaw cycles that could potentially damage the analytes.

### 2.4. Downstream Analysis of the Samples

In order to ensure the requisite quantity and quality of analytes in the collected samples, appropriate analyses and sample measurements were carried out in advance.

#### 2.4.1. Protein Quantification

In samples designated for protein analysis, the total protein content per sample was quantified using a colorimetric detection method based on Coomassie Brilliant Blue (Bradford assay). The analyses were conducted in accordance with the manufacturer’s instructions (Coomassie (Bradford) Protein Assay Kit, Thermo Fisher Scientific Inc., Waltham, MA, USA), and the results were obtained using a spectrophotometer (EON™; BioTek Germany, Bad Friedrichshall, Germany).

#### 2.4.2. RNA/DNA Extraction

The extraction and purification of RNA (including miRNA) and microbial DNA from the wound swab samples was conducted using commercially available extraction kits. Prior to the commencement of this study, extensive testing of various methods and combinations was carried out in order to ensure optimal processing. Total RNA (including miRNA) was isolated using the miRNeasy Micro Kit (Qiagen N.V., Hilden, Germany). Here, centrifugation columns and silica membranes are employed to isolate total RNA, including miRNA, from small-volume samples. Microbial DNA extraction from the swab samples was conducted using the DNeasy PowerSoil Pro Kit (Qiagen N.V., Hilden, Germany), a standard column-based method that has been demonstrated to be highly effective in recovering microbial DNA from a wide range of environmental samples as described previously [15].

#### 2.4.3. Quantity and Quality Analysis of Nucleic Acids (RNA/DNA)

The initial quantification of total RNA was conducted using the NanoDrop 2000 c Spectrophotometer (Thermo Scientific™, Waltham, MA, USA). The NanoDrop method was employed to quantify the absorbance of a 1 µL sample in RNA mode at 260 nm and 280 nm, subsequently calculating the A260/280 ratio, which provides an indication of RNA purity. A ratio in the range of 1.8 to 2.0 indicates optimal purity for downstream analysis. To verify the quantification, an additional measurement of total RNA was conducted using a fluorescence-based method with the Qubit™ Flex Fluorometer (Invitrogen by Thermo Scientific™, Waltham, MA, USA).

To assess the integrity and quality of the RNA samples, they were analyzed using fragment analysis by our core facility (Genomics and Transcriptomics Laboratory—GTL) at Heinrich Heine University Duesseldorf (HHU). The Fragment Analyzer 5300, coupled with the FA DNF-472 HS RNA Kit (Agilent Technologies Inc., Santa Clara, CA, USA), was employed for this purpose. The Fragment Analyzer is a capillary gel electrophoresis instrument that produces an electropherogram, which displays both the size and relative abundance of RNA fragments. Additionally, the instrument generates an RNA quality number (RQN), which serves as a quantitative indicator of RNA integrity and an indication of RNA fragmentation. In the case of small RNA or miRNA, a short nucleotide length (typically <200 nt) is expected in comparison to long non-coding RNA or mRNA, which are typically >200 nt long. 

Quantification of the amount of double-stranded DNA prior to metagenomic sequencing for microbiome analysis was performed using fluorescence-based measurement with the Qubit™ Flex Fluorometer (Invitrogen by Thermo Scientific™, Waltham, MA, USA).

#### 2.4.4. Multiplex Immunoassay

A multiplex immunoassay was employed to determine the concentration of several cytokines, chemokines, proteases and growth factors in small sample quantities in parallel. For this purpose, the Luminex MAGPIX system with xMAP^®^ technology (DiaSorin S.p.A., Saluggia, Italy) was utilized in conjunction with specifically compiled Mix and Match ProcartaPlex™ Multiplex Immunoassay Kits (Thermo Scientific™, Waltham, MA, USA). The sample preparation was conducted in strict accordance with the manufacturer’s protocol.

In this bead-based detection method, the sample is mixed with pre-fabricated colored, magnetic beads in a 96-well plate, which were previously coated with specific capture antibodies for the analytes to be detected. Each bead color is specific for a single analyte. Following the incubation and binding of the analytes to their specific antibodies, biotinylated detection antibodies are added to the mixture, which in turn bind the analyte. This results in the formation of an analyte–antibody sandwich complex. Finally, streptavidin–phycoerythrin (PE), a light-excitable substance, is added, which in turn binds to the biotinylated detection antibodies. Subsequently, the aforementioned complexes are subjected to analysis on the Luminex MAGPIX. The beads are aligned in the wells using a magnet and exposed to lasers of varying wavelengths. A red laser (635 nm) is employed to excite the colored beads and to identify the color of the analyte to be measured. A green laser (532 nm) is employed to excite the bound streptavidin–PE complexes, whereby the intensity of the streptavidin–PE signal is directly proportional to the concentration of the specific analyte and can therefore be quantified. The measurement is conducted with the aid of a charge-coupled device (CCD) camera. Subsequently, quantification is conducted via the integrated xPONENT for MAGPIX software (version 4.3, DiaSorin S.p.A., Saluggia, Italy), wherein measurements are compared against a standard curve constructed from measured standard quantities of the analytes to be quantified. This method permits the simultaneous measurement of up to 50 analytes in a single sample, whereby an initial sample volume of 50 µL is sufficient. 

A total of 36 different analytes were measured in this study, including interleukin-6 (IL-6), matrix metalloproteinase-9 (MMP-9), tissue inhibitor of matrix metalloproteinase-1 (TIMP-1), vascular endothelial growth factor (VEGF) and fibroblast growth factor-2 (FGF-2).

#### 2.4.5. Proteomics

Samples were analyzed by bottom-up shotgun proteomics utilizing a nano liquid chromatography system (nLC) coupled to a quadrupole-orbitrap mass spectrometer (Orbitrap Exploris™ 480, Thermo Fisher Scientific, Bremen, Germany) running in data-dependent acquisition (DDA) mode. 

The sample preparation process comprises the following steps: protein extraction, purification, digestion and concentration of the sample. A combination of the S-Trap method and the STAGE-Tip method was employed to address the heterogeneous (and occasionally low) protein output of the samples and the presence of MS-interfering substances in the samples. The S-Trap™ Mini (100–300 μg/mL) or Micro Spin (<100 µg/mL) column digestion protocol (ProtiFi LLC, Fairport, NY, USA) was employed, in accordance with the manufacturer’s instructions, depending on the protein concentration present in the sample. Subsequently, the samples were alkylated and reduced, after which the proteins were precipitated by the addition of a protein binding buffer from the kit. Subsequently, the sample was applied to the S-Trap column and centrifuged, whereby the proteins were bound in the column. Following several washing steps for purification, a mass spectrometry-grade trypsin/Lys-C mix (Promega Corporation, Madison, WI, USA) was added to the column for overnight in-column protein digestion. This was incubated to cleave the proteins into peptides, specifically at the carboxyl side of lysine and arginine residues. This is important for the correct recognition of peptide fragments in the mass spectrometry database. Subsequently, the peptides were released from the column through a series of elution steps, utilizing the digestion buffer, MS-grade water and finally, 0.1% acetic acid in Aqua dest. (for hydrophobic peptides), and were then pooled into a single sample. 

In order to further purify the sample (i.e., to remove salts and detergents), the STAGE-Tip protocol was employed as a second step. This procedure is described in greater detail in another publication [16]. In general, a new spin column was prepared by custom manufacturing it from the material of an HPLC column, which consists of silica gel and C-18 material, in a modified pipette tip for loading gels. The sample was then subjected to a series of precise and delicate centrifugation steps, during which impurities are effectively removed. Finally, the column containing the analytes was loaded with a 95% aqueous acetonitrile solution (ACN) with 0.1% acetic acid. Elution was performed with N_2_ compressed air into a micro insert, which was then placed into an MS vial (ND8 glass vial with spring and cap with septum). Subsequently, the sample was concentrated to 10 µL using the Concentrator plus—Centrifuge Concentrator (Eppendorf SE, Hamburg, Germany).

Samples were analyzed on an Orbitrap Exploris™ 480 coupled with an UltiMate™ 3 k nanoHPLC using a Flexion Ion Source (all Thermo Scientific, Waltham, MA, USA). Samples were separated using an Acclaim PepMap C18 column (75 µm inner diameter, 25 cm length, Thermo Fisher scientific, run in column oven at 40 °C) on a 240 min gradient running 0.1% formic acid (eluent A) against 95% acetonitrile + 0.1% formic acid (eluent B). Samples were loaded in reverse flush setup onto a precolumn (Pepmap 5 mm cartridge, Thermo Scientific) with 5 µL/min flow of A. Afterward, samples were separated with a flow of 300 nL/min starting at 5% B, up to 32% B in 190 min, up to 99% B in 20 min, followed by washing and re-equilibration to initial conditions. The mass spectrometer was run in DDA with 3 s cycle time consisting of an MS1 scan at 120 k resolution in a mass range of 350 to 1200 *m*/*z*, followed by MS2 scans at 15 k and 30% normalized collision energy. Dynamic exclusion was set to 30 s.

Afterward, samples were analyzed in MaxQuant v2.2 [17] using standard parameters with LFQ option and match between runs option enabled between samples from the same analytical group enabled.

#### 2.4.6. sRNA Sequencing (sRNA-Seq)—miRNA

Following the assessment of the sample quality for an exploratory sRNA-seq study of miRNA in wounds as satisfactory, this study was conducted in the Genomic and Transcriptomic Laboratory (GTL) of the Biological–Medical Research Center (BMFZ) of Heinrich Heine University Duesseldorf. The sequencing process was comprised of a series of discrete steps. Following the successful extraction of total RNA, including miRNA, a cDNA library was created. In this process, the miRNA is transcribed into its complementary DNA sequence (cDNA). This is conducted using a reverse transcriptase process, enabling greater stability during the sequencing process. The QIAseq miRNA Library Kit (Qiagen N.V., Hilden, Germany) was employed for this purpose. A unique molecular identifier (UMI) was assigned to each microRNA throughout the process. These short sequences serve as molecular barcodes that mark each molecule in a library. The use of UMIs can enhance the accuracy and precision of sequencing results, as well as facilitate the correction of errors that may occur during the process. UMIs facilitate the identification of variants by reducing false-positive calls and distinguishing true variants from errors. Subsequent sequencing was conducted on the Illumina NextSeq 2000 System (Illumina Inc., San Diego, CA, USA). The objective was to achieve a total sequencing depth of approximately 30 to 60 million reads per sample. The more intricate process of the complex sRNA-Seq procedure has been described in detail in another source [18].

Subsequently, a bioinformatics analysis was conducted. Demultiplexing and adapter trimming were conducted using BCL Convert (version 3.10.11; Illumina Inc., San Diego, CA, USA) and CLC Genomics Workbench (version 22.0.2; Qiagen N.V., Hilden, Germany) was employed for UMI handling. The sequencing reads were annotated against the miRBase database (release v22 [19]) in order to identify the measured miRNAs. Further statistical analysis of the generated miRNA read counts was carried out using the DESeq2 package in R (R version 4.3.2 [20]).

#### 2.4.7. Metagenomics—Microbiome

A metagenomic analysis of the swab samples was performed to determine the wound microbiome using nanopore sequencing technology. This process entails the sequencing of long segments of the entire genome of the microorganisms present in the samples. The method allows for the detection of not only cultivable microorganisms but also all microorganisms present based on microbial DNA. Following DNA extraction and quality control, the workflow involved the creation of a DNA library for sequencing, with the objective of characterizing the microbial communities present. The Native Barcoding Kit 24 V14 (SQK-NBD114.24; Oxford Nanopore Technologies, Oxford, UK) was utilized to generate the library. Unique barcode adapters were attached to the DNA molecules to differentiate actual identifications from errors in processing and sequencing. 

The sequencing was conducted using the PromethION with R10.4.1 nanopores and V14 chemistry, employing the nanopore technology developed by Oxford Nanopore Technologies (Oxford, UK). This technology is a rapid and precise method for sequencing nucleic acids, renowned for its capacity to generate long reads, which are particularly suited to metagenomic studies. The method is based on an enzymatic process, in which double-stranded nucleic acid is guided to a pore, i.e., nanopore fixated in a membrane on a flow cell and a single strand is pulled through the pore for sequencing. Each nanopore functions as a biosensor, connected to a channel and sensor chip. The current flowing through the nanopore is measured. Single-stranded DNA molecules are driven through the nanopore by an applied gradient. The current flow is repeatedly interrupted as it passes through the nanopore, resulting in a readable electrical signal pattern. This is then decoded with the help of bioinformatic algorithms (“basecalling”), which are used to translate the electrical signal pattern into DNA base sequences. A detailed account of nanopore sequencing technology can be found in Wang et al. [21].

After basecalling, sequence data were subjected to a bioinformatic workup. Initially, the raw sequencing data underwent quality control to remove adapters and inferior reads. Subsequently, a classification of the metagenomic samples was conducted using appropriate bioinformatics tools. The specifics of the bioinformatics analysis procedure are outlined in the statistics section and have been described in detail elsewhere [15].

### 2.5. Statistical Analyses

The present study employed an exploratory approach to evaluate the applicability of state-of-the-art analytical methods to wound swab samples for the purpose of investigating the complex micro-environment of chronic wounds. Consequently, primarily non-confirmatory descriptive statistics were employed. When appropriate, protein, RNA and DNA quantities, as well as the number of identified proteins and miRNAs, were presented as median values with a range in mg/mL (protein) or ng/µL (DNA/RNA), respectively. The data were subjected to analysis using a variety of statistical and bioinformatic approaches, each of which was tailored to the specific methods and objectives of the analysis. 

The results of protein, RNA and DNA quantification and the multiplex immunoassays were analyzed using GraphPad Prism (version 10.2.3; GraphPad Software LLC, Boston, MA, USA). For the latter, the values are presented as the mean ± standard error of the mean (SEM). The scale (in pg/mL) was adjusted to a logarithmic scale (base 10) for enhanced visualization, given that different reference scales apply for each analyte. The statistical significance of the observed differences was evaluated through the implementation of multiple unpaired *t*-tests, with a Holm–Sidak post-hoc test for multiple comparisons.

The results of the proteomics analyses were analyzed using the Perseus software (version 1.6.15.0 [22]) and InstantClue (version 0.12.2 [23]). The data were prepared by initially filtering the dataset based on valid values (70% valid values in at least one group) and performing a log2 transformation. Subsequently, missing data were imputed using the “Minimum Probabilistic Imputation” (MinProb Imputation) approach via the R package “ImputeLCMD” (Imputation for Left-Censored Missing Data). The normal distribution was visually inspected using histograms, and due to the left-skewed nature of the data, normalization was performed using quantile normalization. The statistical analysis of differentially expressed proteins between acute and chronic wounds was conducted using a two-sample *t*-test, with the type I error (false positive results) controlled using the false discovery rate (FDR ≤ 0.05). Principal component analysis (PCA) and volcano plots were employed to visualize the results of the significantly differentially expressed proteins. 

The miRNA raw count data were analyzed in R (R version 4.3.2 [24]) using the DESeq2 package [20]. The primary statistical analyses were descriptive in nature, involving the creation of a principal component analysis (PCA) and the analysis of differentially expressed miRNAs between healing and non-healing wounds in the sample cohort using a volcano plot. The Benjamini–Hochberg (BH) method was employed to control the false discovery rate (FDR), which represents the proportion of false positives among all positive results. A *p*-value of 0.05 was selected as the threshold for identifying miRNAs with statistically significant changes in expression. The threshold for differential expression was set to a fold change of at least 2. To explore the principal components analysis (PCA) results and investigate the main loadings, a biplot was constructed to visually represent the results. Additionally, significant differences in the normalized miRNA counts were visualized using a count plot.

For metagenome analysis, initial quality cutoffs were applied. A taxonomic classification on the genus level for the reads was performed using Kraken 2 [25], and abundances were approximated using Bracken [26]. The results were verified using mapping (minimap2). A detailed description of the bioinformatic process can be found in Spohr et al., 2024 [15]. To provide a visual representation of the microbial species present in the wound swabs, stacked bar charts were generated using the Python-based Altair package (version 5.3.0 [27]).

## 3. Results

### 3.1. Quantity and Quality—Protein/RNA/DNA

**Figure 2:**
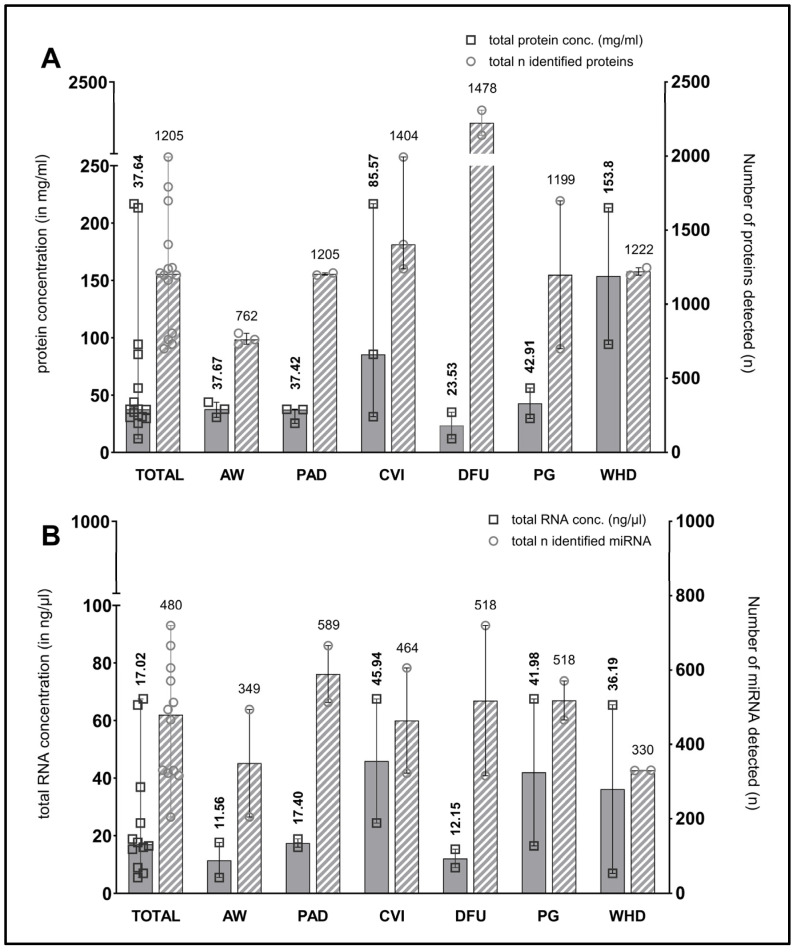
Measured total protein and RNA concentration in samples and number of identified proteins/miRNA in wound swab samples. The figure shows in (**A**) the median measured total protein concentration (in mg/mL on left y-axis; filled bars) in the swab samples in total (“TOTAL”, n = 15) and grouped by entity including measured range. Counts of median identified proteins across all samples (“TOTAL”) and in individual entities are plotted on the right y-axis (striped bars). Median counts for each group are displayed above the bar graphs. (**B**) shows the concentration of total RNA (median + range in ng/µL on left y-axis; filled bars) and the counts of identified miRNA across samples (right y-axis; striped bars) in the same manner as (**A**). (AW—acute wounds; PAD—peripheral arterial disease; CVI—chronic venous insufficiency; DFU—diabetic foot ulcer; PG—pyoderma gangrenosum; WHD—postoperative wound healing disorder).

The median total amount of proteins present in the wound swab samples was 37.64 mg/mL (11.99–216.80). The quantity of protein exhibited considerable variability across the entities, with notable variation observed within the sampled wound types of the sample cohort. Nevertheless, all wound swabs yielded at least 10 mg/mL of protein, which is sufficient for multiple downstream analyses. Figure 2A illustrates the distribution of protein amounts between the entities, as well as the overall distribution within the total sample cohort (n = 12).

Figure 2A also depicts the median numbers of proteins identified through mass spectrometry-based proteomics (plotted on the right y-axis). Only those proteins identified with high confidence following bioinformatic processing were considered. The median number of identifications across all samples is 1205, with a range between 700 and 1994 in the individual samples. Most proteins were identified in venous leg ulcers in patients with chronic venous insufficiency (CVI) and in postoperative wound healing disorders (WHDs), which correlates with both the total amount of protein and the amount of exudation observed in clinical practice in the wounds sampled.

The results of the determination of the amount of extracted total RNA (including small RNA/microRNA) and DNA exhibited heterogeneity across different samples. In most cases, the RNA concentration determined using the Qubit assay was comparable to that obtained with the NanoDrop method, exhibiting a similar trend. In some instances, however, the quantity of RNA material obtained from the swab samples was insufficient for quantification using the Qubit method. Additionally, some samples demonstrated a diminished quality in terms of degradation, as evidenced by an A260/A280 ratio below 1.8. Nevertheless, sufficient concentrated material was obtained for the planned miRNA-Seq analyses. With regard to quality, a somewhat reduced quality was accepted in these initial exploratory analyses in exchange for the possibility of identifying novel miRNAs in some samples. The mean total RNA yield from wound swab samples was 17.02 ng/µL, with a range of 5.53 to 67.51 ng/µL. Figure 2B presents the median amounts of total RNA in the sample cohort and across entities, as well as the median number of identified miRNAs per entity and in the entire sample cohort (plotted on the right y-axis).

A comparable pattern was identified in the context of DNA extraction, albeit with the measurement of solely double-stranded DNA using a Qubit. Furthermore, heterogeneity was observed in the samples. The quantity of DNA extracted differed depending on whether the sample was processed without cells (i.e., solely from the supernatant) or with cells (from the centrifuged pellet). Consequently, a combination of cell pellets and the supernatant was used for metagenomics and utilized for further preparation for sequencing. Consequently, an average of 61.49 ± 61.37 ng/µL of DNA could be isolated from the wound swab samples. This quantity is sufficient for the planned metagenomic analysis and the exclusive detection of double-stranded DNA, as the relevant input material for nanopore sequencing is itself a positive quality control. The quantity of DNA extracted from the swab samples is presented graphically in Figure 6.

### 3.2. Protein Profiling—Multiplex Immunoassay

**Figure 3:**
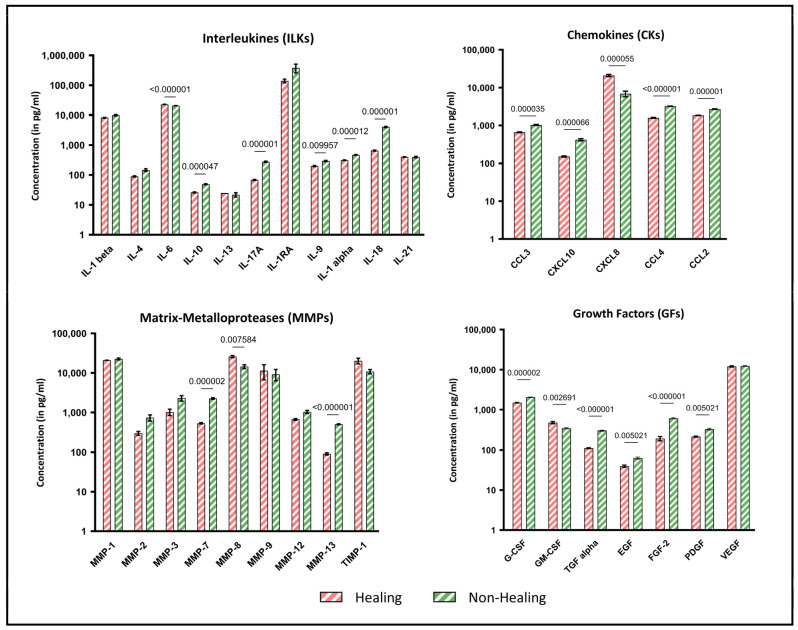
Multiplex immunoassay analyses of wound swab samples for healing and non-healing wounds. Shown are the results of the multiplex immunoassay analyses of the sample cohort grouped by interleukins (ILKs), chemokines (CKs), matrix metalloproteases (MMPs) and growth factors (GFs). The results are shown as concentration (pg/mL) with logarithmic scaling for better visualization. The different concentrations in wounds clinically classified as healing (n = 4) and non-healing (n = 8) were pooled for all entities analyzed and compared statistically. Values are presented as means ± S.E.M. and results were analyzed using multiple unpaired *t*-tests with a Holm-Sidak post-hoc test for multiple comparisons.

The analysis of biomolecular signal transducers, including cytokines, chemokines and growth factors, revealed notable differences between wound swab samples from healing and non-healing wounds. Figure 3 provides an exemplary overview of the holistic local environmental differences between the analytes, with a focus on which of these are significantly different. Several proinflammatory cytokines, including IL-17a (*p* = 0.000001), IL-1alpha (*p* = 0.000012) and IL-18 (*p* = 0.000001), were found to be markedly increased in non-healing wounds. Conversely, the pro-inflammatory cytokine IL-6 was also found to be significantly lower in non-healing wounds (*p* < 0.000001), while the anti-inflammatory IL-10 was significantly increased in non-healing wounds (*p* = 0.000047).

The results for chemokines, matrix metalloproteases and growth factors also demonstrate increased pro-inflammatory indications. While nearly all growth factors are significantly reduced in non-healing wounds (*p* ≤ 0.05), chemokines (CCL-2, -3, -4 and CXCL10), which are particularly active during inflammation, are expressed at higher levels in non-healing wounds. In contrast, CXCL-8, which is also involved in the inflammatory response, is significantly increased in healing wounds in this sample cohort (*p* = 0.000055). These profile analyses provide information about the diffusely regulated immune response to local tissue destruction, pathogen defense and possible healing tendencies. 

Similarly, the analyzed MMPs exhibited a significantly higher concentration in non-healing wounds than in healing wounds. MMP-7 (*p* = 0.000002) and MMP-13 (*p* < 0.000001) exhibited significantly higher values in non-healing wounds. In contrast, MMP-8 was expressed at markedly higher levels in healing wounds in this sample cohort (*p* = 0.0076). This may be indicative of collagen remodeling from earlier types (type III) to the later dominant type I. Tissue Inhibitor of Metalloproteinase 1 (TIMP-1), the antagonist of MMPs, was reduced in non-healing wounds, although the results were not statistically significant in this cohort.

### 3.3. Protein Profiling—Proteomics

The initial exemplary analyses of the proteome data demonstrate the potential of exploratory approaches for diagnostic purposes. In total, over 2907 distinct proteins were identified in the sample cohort (n = 12), with the range of proteins identified with a high degree of certainty per sample being 700 to 1994 proteins. A variety of proteins were identified in the samples, many of which are crucial for cutaneous regeneration. Certain proteins were as expected regarding the sample origin as being defective cutaneous tissue with exposed connective tissue and extracellular matrix. These included relevant drivers and building blocks of tissue regeneration, such as collagen type 1–3, matrix metalloproteases (e.g., MMP-1, -2, -8, and -9), cathepsins, laminin, keratin and others. Many of such proteins thereby represent a potential common baseline identifying the sample as a “wound sample”, useful for baseline adjustment or quality controls. Additionally, immune defense proteins, including antimicrobial peptides (e.g., alpha-defensin or cathelicidin), enzymes (e.g., myeloperoxidase), complement factors and immunoglobulins were identified, representing aspects of the human defensive and reparative system active during reparative processes. The degree to which the system is active and the specific players involved are the main areas of interest, as inflammatory processes are a major concern in wound healing. A multitude of other proteins pertinent to various human biological processes have been identified, thus enabling an in-depth examination of the proteome of chronic wounds by wound swabbing. 

**Figure 4:**
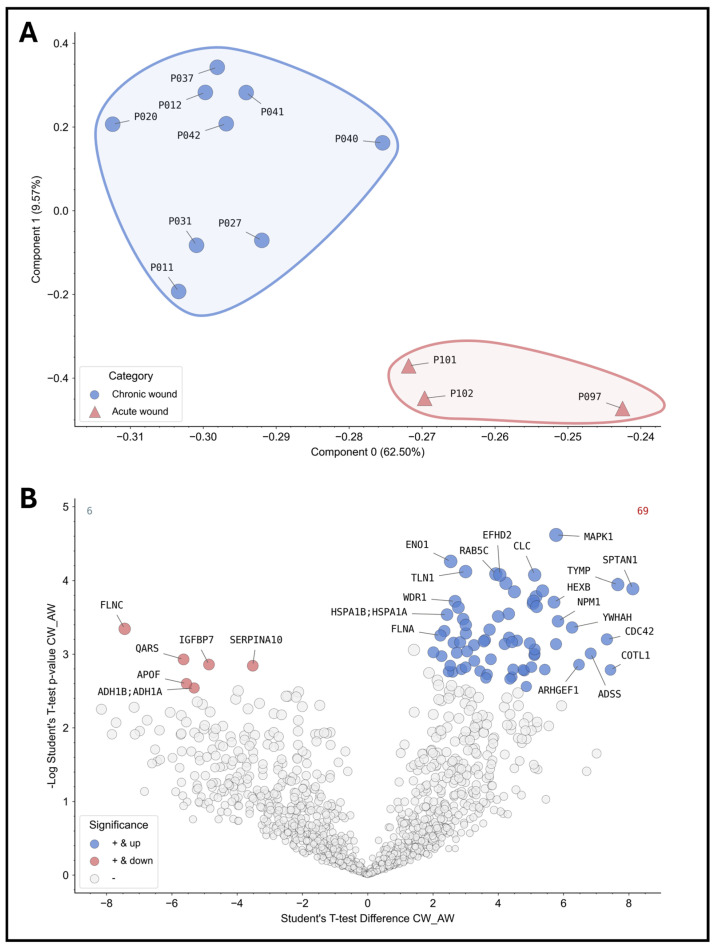
Proteomic analyses of the wound swab samples for acute and chronic wounds. The results of the proteomics analyses of the sample cohort are shown. On the top (**A**), the patient samples were grouped into acute (red, n = 3) and chronic wounds (blue, n = 9) according to the study definition and analyzed using PCA. The analysis demonstrates the feasibility of differentiating acute and chronic wounds based on their inert protein signature showing distinct clusters. Thus, differentiation between acute and chronic wounds based on their “proteome fingerprint” for diagnostic purposes is possible in principle. The volcano plot below (**B**) depicts differentially expressed proteins or proteins present in greater or lesser relative abundance in comparison between acute and chronic wounds (values were z-score transformed for visualization). All colored proteins above the cut-off (FDR = 0.05) are significantly up- (right in blue, 69 proteins in total) or down-regulated (left in red, 6 proteins in total) in chronic wounds. As an example, the most differentially expressed proteins were annotated with their coding gene names (a complete list is provided in the Supplementary Files in Supplementary Table S1).

For instance, principal component analysis (PCA) can be employed to effectively differentiate between various subgroups of wound samples and to distinguish between wound types, stages and entities through clustering. Figure 4A illustrates an example of principal component analysis (PCA) clustering across sampled wounds. The underlying protein signature allows for the distinction between acute and chronic wounds in the initial analysis of the samples obtained. Subsequent analysis of significantly differentially expressed proteins (Figure 4B) revealed a total of 75 differentially abundant proteins in comparison between acute and chronic wounds, with 6 significantly down-regulated proteins (e.g., FLNC—“filamin-C”; IGFBP7—“Insulin Like Growth Factor Binding Protein 7”; SERPINA10—“Protein Z-dependent protease inhibitor”) and 69 significantly up-regulated (e.g., CLC—“galectin-10”; MAPK1—“Mito-gen-activated protein kinase 1”; ENO1—“Alpha-enolase”). The results will provide deeper insights into the processes that are disturbed in chronic wounds and which potential biomarkers should be selected for further analyses.

### 3.4. SmallRNA Seq—miRNA

As with the proteome data, the initial miRNA sequencing analyses yielded promising results. In total, a median of 480 distinct miRNAs were identified in the sample cohort with adequate expression levels (range 205–720), of which, 183 miR-NAs were detected in all samples. As regulators of gene expression and potential biomarkers for different phases and stages of wound healing and tissue regeneration, miRNA analysis provides further valuable insight into the environment of healing and non-healing wounds. 

**Figure 5:**
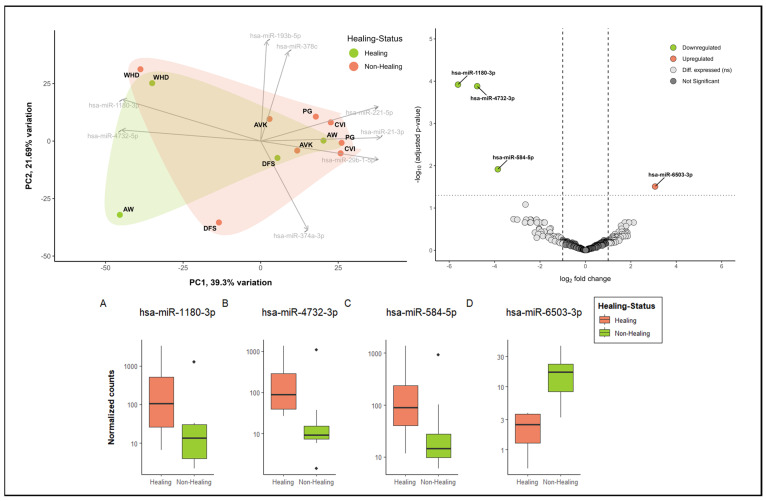
Exploratory analysis into the differential miRNA patterns of healing (n = 4) vs. non-healing wounds (n = 8). In the upper left corner, a biplot demonstrates the dimensional reduction of the dataset using a PCA analysis, whereby about 39% of the variation between categories (healing vs. non-healing) is explained by component 1 and about 22% by component 2. The top 8 driving miRNAs are depicted as loadings and the directions they are driving the PCA plot. Healing and non-healing wounds demonstrate major overlaps in clustering in this sample cohort; however, specific aspects of healing wounds can also be observed. Additionally, cluster formation of specific entities (here, WHD in the upper left corner) can be observed. Differential expression analysis of miRNA expression in wound swab samples from healing and non-healing wounds in the upper right corner (volcano plot) shows significantly up- and down-regulated miRNA in comparison between clinically healing and non-healing wounds. All colored miRNAs above the cut-off (FDR = 0.05) are significantly up-regulated (right in red, one miRNA) or down-regulated (left in green, 3 miRNA) in non-healing wounds compared to healing wounds. The differentially expressed miRNAs were annotated by name and their differences in normalized count were plotted in individual count plots (**A**–**D**) at the bottom.

As an illustration, Figure 5 presents the initial exploratory insights into miRNA patterns in healing versus non-healing wounds. The principal component analysis (PCA) reveals the presence of distinct clusters, with wound healing disorders (WHDs) exhibiting a notable separation from other entities. However, no discernible differentiation between healing and non-healing wounds can be observed in the initial sample cohort (Figure 5—PCA plot upper left corner). The two states are largely overlapping in the clusters. Nevertheless, there are also miRNAs that differentiate healing wounds from non-healing wounds. These miRNAs, hsa-miR-1180-3p and has-miR-4732-5p, are two of the top loadings in the PCA. Additionally, both miRNAs are among the three most significantly down-regulated miRNAs in non-healing wounds, further substantiating their association with the healing process (Figure 5A,B). The volcano plot (Figure 5) and the normalized count plots (Figure 5C,D) depict two additional differentially expressed miRNAs in comparison between wound swabs from healing and non-healing wounds. These are hsa-miR-584-5p (down-regulated in non-healing wounds) and hsa-miR-6503-3p (up-regulated in healing wounds). This illustrates the value of in-depth analysis of the differences between healing status and wound entities. These analyses demonstrate the feasibility of utilizing material obtained from wound swabs for the analysis of miRNA, thereby providing insights into the regulatory functions of miRNA in wounds.

### 3.5. Metagenomics—Microbiome

**Figure 6:**
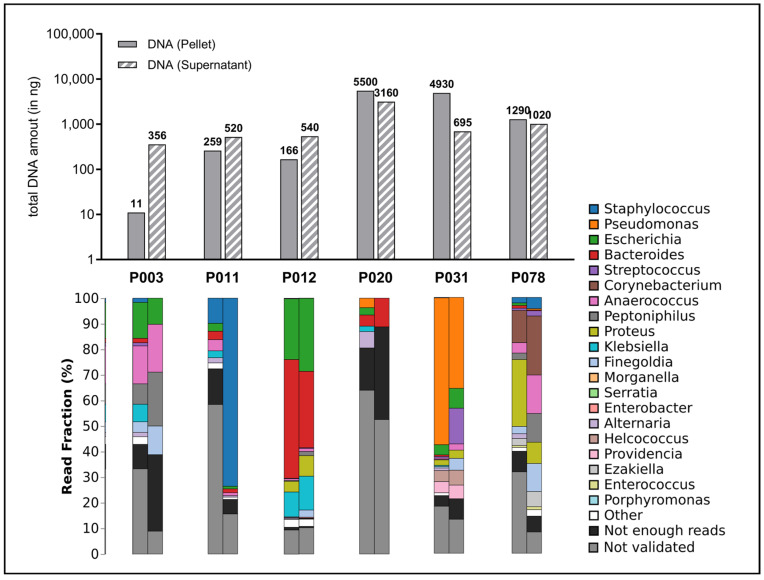
Amount of total DNA extracted and distribution of detected microbial species. The upper portion of the graph depicts the total quantity of extracted DNA (in nanograms) per sample, presented as bar charts. Here, both the quantities of the preserved supernatant (striped bars) and the quantity in the cell pellet produced during the work-up (filled bar) were measured since it was assumed that a significant proportion of microorganisms were present in the debris pellet. The lower section of the graph displays the fractional distribution (in percentage) of the top 20 identified genera in the swab samples from the wounds (cell pellet on the left, supernatant on the right), presented in stacked bar charts.

The sequencing of the microbiome of wound swabs provides a deep insight into the diversity and relative abundance of the different microbial taxa in the respective wound sites (Figure 6).

The use of a sequencing method, nanopore technology, which produces particularly long read lengths, should be emphasized here. In contrast to methods that produce short sequences (e.g., Illumina) or are based on a purely cultural differentiation of pathogens, this method provides a more detailed picture of the microbiome. Specifically, species frequently associated with wound infections (*Staphylococcus* spp., *Pseudomonas* spp., *Streptococcus* spp.) were detected. In addition, some samples also showed pathogens that are less commonly associated with wound infections (*Proteus* spp., *Klebsiella* spp.) and some anaerobes (*Bacteroides* spp., *Anaerococcus* spp., *Finegoldia* spp.), whose relevance for the stagnation of wound healing, the development of wound infections and as members of microbial communities (biofilm) must be clarified in future studies.

## 4. Discussion

The correct and adequate diagnosis of the underlying pathology of a chronic wound at the earliest possible stage, as well as the identification and monitoring of negative influencing factors, is the essential basis for optimal treatment planning [1,9]. However, further local therapy for chronic and non-healing wounds is often protracted during treatment and is largely based on the experience of the practitioner. There are few to no valid and proven diagnostic approaches for analyzing the complex local chronic wound micro-environment or underlying molecular deficits that would facilitate specific therapeutic interventions or generally reflect the success of local therapy for adequate and objective monitoring. Beyond the histological analysis of a tissue biopsy, which for various reasons (indication, resources, relevance) is only used in cases of prolonged stagnant healing or suspected atypical or malignant wounds, and the routine local swab sampling for mainly cultural determination of potential microbial pathogens, analyses of the local wound micro-environment are thus far rarely performed in routine clinical practice. The potential of various individual approaches, however, has been sporadically postulated in the past as an opportunity for individualized and targeted therapy and promising work has been published in this regard [6,28,29]. Possible diagnostics and local control parameters range from pH value [30], fluorescence imaging [31] and hyperspectral imaging [32,33] to microbiome analysis [13].

In this study, we focused on the in-depth investigation of the utility of local wound swabs beyond their established microbiological diagnostic application. The wound swab is a universally known, accessible and easy-to-use tool that is ubiquitous in everyday medical practice. It is inexpensive, easy to use and not painful for patients. In contrast to many other modern diagnostics outlined above, it is therefore ideal for obtaining valuable samples from acute and chronic wounds. The vision is to identify biomolecular markers, patterns and signatures for in-depth and at some point, bedside diagnostics of pathological processes in wound healing and for obtaining therapeutic monitoring parameters to objectify local wound therapy. Earlier studies and first commercial approaches (e.g., WOUNDCHEK™) have pursued partially similar approaches to carry out immunomarkers [6,12,28,29,34], proteome [2,3,4,5] and microbiome [13] analyses from wound swabs and thus achieved promising preliminary success, contributing relevant observations to the knowledge base on the regenerative process of wound healing. Studies based on analyzing wound-healing parameters from wound exudate for example identified some of the first potential biomarkers that might be capable of differentiating healing from non-healing wounds over the treatment course (e.g., MMP-13 or GM-CSF [29]) or indicate wound infections that have not yet shown clinical manifestation [34]. In the future, such potential point-of-care diagnostics can contribute to an objectively informed, targeted and individualized wound management approach. Newly proposed local treatment approaches, such as new antiseptic agents, cold atmospheric plasma, protease-modulating or inflammation-reducing local wound treatment could be administered based on biomolecular profiles, reacting specifically to a monitored pathologic process instead of applying treatments simply on the fact that a wound is not healing properly or subjectively judged to persist in a certain state. Also, monitoring parameters might be used as prognostic and predictive markers to estimate healing trajectories (e.g., MMP-13 or GM-CSF). 

Building on these approaches, we initiated the “Wound-BIOME” project, which aims to further develop our understanding of the complex wound micro-environment by analyzing and unifying the biological levels involved (proteome, microbiome, transcriptome, etc.) and identifying robust diagnostic parameters based on wound swabbing that can support more objective local therapy and allocation of meaningful interventions to the correct recipients, reducing “trial and error” approaches in the future. In the study presented here, we outline the methodological work behind this with the testing of a simple swab protocol for sample collection that can be carried out on an outpatient as well as clinical basis and the results of the feasibility analyses of a sample cohort. 

The sample collection protocol shown in Figure 1 is ultimately no more complicated or time-consuming than the correct swab collection for microbiological analysis, and no pre-processing or complicated freezing procedure is required by the primary user. The equivalent success of this protocol compared to regular microbial analyses is already demonstrated by the metagenomic analyses performed here. Compared to the sole culture approach, however, the entirety of the microorganisms present can be detected on the basis of genome sequencing, regardless of their cultivability, and a differentiated picture of the prevailing microbial load can be generated (Figure 6). The quantity and quality analyses carried out here show that sufficient intact DNA material can be obtained from regular swabs to generate high-quality analyses. Regarding the microorganisms identified in the sample cohort, the observations align with the expectations. Additionally, less frequently observed bacteria with routine microbiological testing were also identified, which yields the questions if this is to be attributed solely to the advanced technique (nanopore) and whether this observation has further implications for managing bioburden and treatment of chronic wounds. Future in-depth work will be necessary to aim to answer this question. Potentially, however, the newfound deeper insights into the microbial burden could inform the usage of specific antimicrobial and antibiotic treatments on a more in-depth level, contributing to addressing the global issue of antimicrobial resistance or decisions regarding advanced microbial diagnostics in certain non-healing wounds.

The same proof of feasibility applies to the quantity and quality of isolated protein and RNA material (Figure 2). The successful results obtained in the downstream analyses of the proteome and miRNA expression impressively demonstrate the value of such analyses for the future of diagnostic and therapeutic management of people with chronic wounds. Distinct protein “fingerprints” of individual wound entities (here, e.g., acute and chronic wounds; Figure 4) enable potential differentiation between stages of healing as they correlate with certain phases of regeneration. This may allow for improved diagnostics regarding uncertainty in entity specification or underlying pathophysiology driving chronicity in individual wounds, thereby improving the management of interventions applied. Such signatures could also help to clarify prevailing pathological processes in mixed ulcerations or provide biomarkers or marker networks (Figure 5) that indicate stages of the healing process in order to monitor progress and efficacy during treatment. To date, the most researched area of inflammatory immunomarkers for monitoring pro- or anti-inflammatory or proteolytic milieu shifts was also successfully integrated in this study (Figure 3). It could be demonstrated that they can be addressed just as well as in previous studies and other analyses tackled in this work. Our results of this feasibility study thereby show comparable results and trends as previous publications [6,12,29] with increased expression of pro-inflammatory cytokines and proteolytic MMPs with reduced levels of growth factors in non-healing wounds. The demonstration of equivalent results supports the validity of our approach. In addition to the simple sample collection and handling, the required sample quantity is low. In the analyses shown here, sample quantities of 50–200 µL (each for proteins and RNA/DNA) were ultimately sufficient for all presented analyses. 

Naturally, this is only the start of more extensive and in-depth research work still to be performed. The presented study, however, demonstrates that the approach can generate valuable, useful and valid results already in comparably small sample cohorts (n = 12). However, based on the preliminary nature of the data, certain limitations of the current results must be considered. In addition to the small number of samples on which these initial results are based, it must be noted that in many of the methodological areas described, the analysis of wound exudate and specifically swab samples breaks new ground or at least very little previous work exists. Therefore, new experience is gained, and unexpected problems arise with each step from sample collection to data analysis that need to be met along the way. Since the bioinformatic analyses can only be as good as the biological samples from which they derive, larger cohorts with rigorous analyses must demonstrate the robustness of the collection strategy and processing procedure in the future. Also, the sample cohorts in this initial feasibility study differed across analysis platforms, which makes the presented preliminary data only interpretable for each field on its own. The results of differential analyses shown here are therefore initial, exploratory results that need to be reproduced, revisited and validated in a larger context. The next steps will be to apply and verify preliminary findings and developed workflows in the entire cohort of the “Wound-BIOME” project (n = 120 patients). The analyses of protein, RNA and microbiome signatures, which were only outlined here as examples, will be explored in greater depth to not only describe recognizable patterns but also to identify potentially relevant marker sets for predictive application and improve our understanding of the chronically dysregulated wound micro-environment. Another important and highly interesting step will be the attempt at a bioinformatic unification of the respective biological levels and planes investigated. Using multi-OMICS approaches in order to not only glimpse at individual layers but to try to connect the complex processes in their spatial and temporal interacting resolution. Therefore, the OMICS analyses described above are carried out in all patients included in the project to be able to superimpose these layers. This is quite a challenge in the still-young field of systems biology, but the developments of recent years made such in-depth, multi-layered analyses into the regenerative environment of chronic wounds possible for the first time. Finally, the economic aspect must not be ignored. While the collection and storage of samples is generally inexpensive and less complex and the field of OMICS analyses is constantly expanding with options becoming more affordable, facilities that have the necessary expertise and resources to analyze complex signatures are so far mostly exclusive at university centers and core facilities. It is therefore crucial to pursue further reductions in the complexity of future analyses and to test standardized laboratory algorithms on a range of platforms to render the potential diagnostics presented here suitable for routine clinical practice.

## 5. Conclusions

In summary, with this study on the usability of classical wound swabs using a defined sampling procedure, we were able to demonstrate that swab samples from acute and chronic wounds provide sufficient high-quality material to perform a variety of state-of-the-art OMICS and multiplex analyses to investigate the wound micro-environment as a first step of the “Wound-BIOME” project. The results obtained from the sample cohort impressively demonstrate the potential to detect inflammatory signatures, differentiate subgroupings and generate potential biomarker sets as well as analyze microbial communities within the wounds. The potential of the “WoundOMICS” approach for objective diagnostic monitoring parameters for an individualized wound therapy to complement and expand clinical–practical expertise instead of a “trial-and-error” approach is clearly recognizable, and further studies within the “Wound-BIOME” project will pursue this potential.

## Figures and Tables

**Table 1:** Inclusion and exclusion criteria of the “Wound-BIOME” project.

Inclusion Criteria	Exclusion Criteria
Patient ≥ 18 years	Patient < 18 years
Wound area ≥ 1.5 cm^2^	Wound area < 1.5 cm^2^
Presence of a wound which either - **Persists for more than 8 weeks** under adequate therapy (“**chronic**”) **OR** is considered chronic due to their underlying disease - **No shorter** than 24 h and **no longer** than 6 days after traumatic event/surgical intervention (“**acute**”)	Dry necrosis
	Pregnancy and breastfeeding
	Malignant genesis of the wound (e.g., ulcerating soft tissue tumor)

**Table 2:** Characteristics of the cohort and analyses performed per sample. The underlying etiology of the wound, the clinical healing status (H = healing, NH = non-healing), the recruiting clinical center and the downstream analyses to which the samples were submitted are listed.

ID	Etiology	Status	Center	Immunoassay	Proteome	miRNA	Microbiome
P003	DFU	NH	UWH	✓	-	✓	✓
P011	PAD	NH	UWH	✓	✓	✓	✓
P012	PAD	NH	UWH	-	✓	-	✓
P020	CVI	NH	UWH	-	✓	-	✓
P027	WHD	NH	UKE	✓	✓	✓	-
P031	CVI	NH	UKE	✓	✓	✓	✓
P037	PG	NH	UKE	✓	✓	✓	-
P039	PAD	NH	UKES	✓	-	✓	-
P040	PG	NH	UKES	✓	✓	✓	-
P041	WHD	H	UKES	✓	✓	✓	-
P042	CVI	NH	UKES	✓	✓	✓	-
P078	DFU	H	UKES	✓	-	✓	✓
P097	AW	H	UKD	✓	✓	✓	-
P101	AW	H	UKD	-	✓	-	-
P102	AW	H	UKD	✓	✓	✓	-

DFU—diabetic foot ulcer, PAD—peripheral arterial disease, CVI—chronic venous insufficiency, WHD—postoperative wound healing disorder, PG—pyoderma gangrenosum, AW—acute wound; UWH—Witten/Herdecke University, UKE—University Medical Center Hamburg-Eppendorf, UKES—University Medical Center Essen, UKD—University Medical Center Duesseldorf.

**Table 3:** Key points of the sampling procedure. Illustration of the main steps of sample collection. Critical points in order to prevent significant sample falsification are highlighted.

Sample Collection and Preservation	
1	Removal of dressing material from wound
	🡺 Only use sterile physiological 0.9% NaCl or ringer solutions to loosen coatings and dressings before taking samples. (to prevent sample falsification)
2	Mechanical cleansing of the wound and removal of slough if necessary(to sample the actual wound bed)
	🡺 No sharp debridement to avoid bleeding or, in the event of unwanted bleeding, complete hemostasis first. (prevention of sample contamination by blood components)
3	Swabbing according to “Essen Rotary” until swab tip visibly saturated
	🡺 Use of max. 5 mL sterile neutral solution (e.g., 0.9% NaCl) for moistening the wound bed before sampling possible.
4	Place saturated swab in transport container, break off at breaking point and close container
5	Freeze to at least −18 °C within 2 h and to −80 °C within 24 h

## Data Availability

The raw data supporting the conclusions of this article will be made available by the authors upon request.

## Supplementary Materials

The following supporting information can be downloaded at: https://www.mdpi.com/article/10.3390/biomedicines12102187/s1. Table S1: Significantly differentially expressed proteins in acute and chronic wounds.
