# Supplementary material for: Assessment and Monitoring of the Wound Micro-Environment in Chronic Wounds Using Standardized Wound Swabbing for Individualized Diagnostics and Targeted Interventions
Source: Biomedicines. 2024 Sep 26;12(10):2187. doi: 10.3390/biomedicines12102187 (PMC11505098; doi:10.3390/biomedicines12102187)
Supplement: Supplementary file 1 [file biomedicines-12-02187-s001.zip › biomedicines-3151785-supplementary.pdf]

Protein name	Gene name	up-/downregulated	q-value
Chloride intracellular channel protein 1	*CLIC1*	**up**	*0.031*
WD repeat-containing protein 1	*WDR1*	**up**	*0.027*
Argininosuccinate lyase	*ASL*	**up**	*0.033*
Alpha-enolase	*ENO1*	**up**	*0.026*
Glucose-6-phosphate isomerase	*GPI*	**up**	*0.045*
Nucleophosmin	*NPM1*	**up**	*0.027*
Protein disulfide-isomerase	*P4HB*	**up**	*0.037*
Beta-hexosaminidase subunit beta; Beta-hexosaminidase subunit beta chain B; Beta-hexosaminidase subunit beta chain A	*HEXB*	**up**	*0.022*
Profilin-1	*PFN1*	**up**	*0.035*
Heat shock protein HSP 90-alpha	*HSP90AA1*	**up**	*0.044*
Heat shock protein HSP 90-beta	*HSP90AB1*	**up**	*0.033*
Pleckstrin	*PLEK*	**up**	*0.044*
Heat shock 70 kDa protein 1B; Heat shock 70 kDa protein 1A	*HSPA1B; HSPA1A*	**up**	*0.032*
78 kDa glucose-regulated protein	*HSPA5*	**up**	*0.036*
Annexin A3	*ANXA3*	**up**	*0.032*
Elongation factor 2	*EEF2*	**up**	*0.029*
Plastin-2	*LCP1*	**up**	*0.028*
Alcohol dehydrogenase [NADP(+)]	*AKR1A1*	**up**	*0.026*
Vinculin	*VCL*	**up**	*0.047*
Thymidine phosphorylase	*TYMP*	**up**	*0.045*
Azurocidin	*AZU1*	**up**	*0.043*
Filamin-A	*FLNA*	**up**	*0.034*
Cathepsin S	*CTSS*	**up**	*0.037*
Moesin	*MSN*	**up**	*0.033*
Mitogen-activated protein kinase 1	*MAPK1*	**up**	*0.016*
Transketolase	*TKT*	**up**	*0.035*
Elongation factor 1-delta	*EEF1D*	**up**	*0.032*
Protein disulfide-isomerase A3	*PDIA3*	**up**	*0.025*
Adenylosuccinate synthetase isozyme 2	*ADSS*	**up**	*0.033*
Leukocyte elastase inhibitor	*SERPINB1*	**up**	*0.032*
Rab GDP dissociation inhibitor alpha	*GDI1*	**up**	*0.030*
Myosin-9	*MYH9*	**up**	*0.044*
Transaldolase	*TALDO1*	**up**	*0.037*
Macrophage-capping protein	*CAPG*	**up**	*0.023*
Ras GTPase-activating-like protein IQGAP1	*IQGAP1*	**up**	*0.032*
Serpin B10	*SERPINB10*	**up**	*0.023*
Ras-related protein Rab-5C	*RAB5C*	**up**	*0.037*

6-phosphogluconate dehydrogenase, decarboxylating	*PGD*	**up**	*0.048*
Rho GDP-dissociation inhibitor 2	*ARHGDIB*	**up**	*0.029*
Cell division control protein 42 homolog	*CDC42*	**up**	*0.028*
Ras-related protein Rab-2A	*RAB2A*	**up**	*0.022*
Ras-related protein Rab-14	*RAB14*	**up**	*0.045*
Transforming protein RhoA	*RHOA*	**up**	*0.030*
Epididymal secretory protein E1	*NPC2*	**up**	*0.031*
Heterogeneous nuclear ribonucleoprotein K	*HNRNPK*	**up**	*0.047*
Guanine nucleotide-binding protein G(I)/G(S)/G(T) subunit beta-2	*GNB2*	**up**	*0.044*
Clathrin heavy chain 1	*CLTC*	**up**	*0.036*
Fatty acid-binding protein, epidermal	*FABP5*	**up**	*0.043*
Adenylyl cyclase-associated protein 1	*CAP1*	**up**	*0.031*
14-3-3 protein eta	*YWHAH*	**up**	*0.028*
Galectin-10	*CLC*	**up**	*0.047*
Interleukin enhancer-binding factor 3	*ILF3*	**up**	*0.035*
Spectrin alpha chain, non-erythrocytic 1	*SPTAN1*	**up**	*0.051*
Coactosin-like protein	*COTL1*	**up**	*0.035*
Septin-6	*SEPT6*	**up**	*0.030*
Major vault protein	*MVP*	**up**	*0.033*
Splicing factor 3A subunit 1	*SF3A1*	**up**	*0.046*
Ras-related protein Rab-11B	*RAB11B*	**up**	*0.023*
Thioredoxin reductase 1, cytoplasmic	*TXNRD1*	**up**	*0.031*
Twinfilin-2	*TWF2*	**up**	*0.031*
Phospholipase B-like 1; Phospholipase B-like 1 chain A; Phospholipase B-like 1 chain B; Phospholipase B-like 1 chain C	*PLBD1*	**up**	*0.031*
Rho guanine nucleotide exchange factor 1	*ARHGEF1*	**up**	*0.034*
EF-hand domain-containing protein D2	*EFHD2*	**up**	*0.044*
Golgi-associated plant pathogenesis-related protein 1	*GLIPR2*	**up**	*0.032*
EH domain-containing protein 1	*EHD1*	**up**	*0.043*
Resistin	*RETN*	**up**	*0.036*
Dipeptidyl peptidase 3	*DPP3*	**up**	*0.035*
N-acetyl-D-glucosamine kinase	*NAGK*	**up**	*0.027*
Talin-1	*TLN1*	**up**	*0.028*
Protein Z-dependent protease inhibitor	*SERPINA10*	**down**	*0.037*
Insulin-like growth factor-binding protein 7	*IGFBP7*	**down**	*0.035*
Filamin-C	*FLNC*	**down**	*0.027*
Apolipoprotein F	*APOF*	**down**	*0.044*
Glutamine--tRNA ligase	*QARS*	**down**	*0.033*
Alcohol dehydrogenase 1B; Alcohol dehydrogenase 1A	*ADH1B; ADH1A*	**down**	*0.047*

Table S1. Significantly differentially expressed proteins in acute and chronic wounds. Proteins and corresponding gene names that are significantly differentially expressed in chronic wounds compared to acute wounds and the direction of fold change (up- or downregulated). The rate of false positive discoveries was controlled at the level of $\alpha < 0.05\%$ using a permutation-based False Discovery Rate (FDR) for multiple comparisons. The q-value corresponding to an adjusted p-value in FDR is reported.
